# Binding of the brain G protein G⍺_o_ to its potential effector RASA3 is promoted by Ca^2+^

**DOI:** 10.1016/j.jbc.2025.110999

**Published:** 2025-12-03

**Authors:** Halie A.S. Bell, Andrew C. Olson, Juliana E. Gentile, TuKiet T. Lam, Michael R. Koelle

**Affiliations:** 1 Department of Molecular Biophysics and Biochemistry, Yale University, New Haven, Connecticut, USA; 2 Keck MS & Proteomics Resource, Yale School of Medicine, New Haven, Connecticut, USA

**Keywords:** heterotrimeric G protein, G⍺_o_, RASA3, GTPase-activating protein (GAP), proteomics, neurotransmitter signaling, protein–protein interaction

## Abstract

G⍺_o_, the alpha subunit of the most abundant heterotrimeric G protein in the brain, mediates signaling by opioids and by many neuromodulators to inhibit neural function. An open question is whether activated G⍺_o_-GTP directly binds to and regulates effector molecules, like all other animal G⍺ proteins, or if it signals solely by releasing Gβ*γ* subunits. Using mouse brain lysates as native source of G⍺_o_ and its potential effectors, we analyzed immunopurified G⍺_o_ protein complexes by mass spectrometry. Pre-activating G⍺_o_ in the lysates with GTP*γ*S resulted in a ∼6-fold increase in the amount of the small G protein GTPase activators RASA3 and RASA2 in the purified complexes, the largest increase among all G⍺_o_-associated proteins, making RASA2/3 candidate G⍺_o_ effectors. Using purified recombinant proteins, we found that RASA3 binds directly to G⍺_o_-GTP*γ*S more strongly than it does to G⍺_o_-GDP. We also found that the addition of Ca^2+^, a second messenger produced by the G⍺_q_ pathway that opposes G⍺_o_ signaling, strengthened binding of RASA3 to G⍺_o_-GDP. A C-terminal fragment of RASA3 containing a predicted Ca^2+^ site was sufficient to bind G⍺_o_, albeit more weakly and without a preference for the activated state of G⍺_o_. We present a model in which RASA3 could mediate G⍺_o_ signaling using two distinct G⍺_o_-binding sites: one on full-length RASA3 that preferentially binds active G⍺_o_-GTP and a second on the RASA3 C terminus that binds inactive G⍺_o_ in the presence of Ca^2+^.

G⍺_o_ is the alpha subunit of the most abundant heterotrimeric G protein of the nervous system, constituting 1.5% of the membrane protein in the brain (1). It mediates the addictive and analgesic effects of opioids as well as signaling by a wide variety of neurotransmitters and neuropeptides to inhibit neurotransmitter release (2). Heterotrimeric G proteins in the brain are activated when a ligand-bound G protein–coupled receptor acts as a guanine nucleotide exchange factor, promoting the exchange of GDP for GTP on the G⍺ subunit and the dissociation of the Gβ*γ* complex from the G⍺ subunit. Both G⍺-GTP and free Gβ*γ* can bind to and modulate the activity of target proteins, known as effectors, to initiate downstream signaling (3). All known effectors for G⍺ proteins bind to the G⍺ switch regions, which change conformation upon exchange of GDP for GTP, allowing these effectors to bind G⍺-GTP in preference to G⍺-GDP (4).

G⍺_o_ is the only G⍺ protein in animals for which no conserved effector is widely agreed upon. G⍺_o_ is in the same G⍺ subfamily as the three isoforms of G⍺_i_, each of which can bind and inhibit multiple adenylyl cyclase isoforms. However, G⍺_o_ has a far weaker ability to inhibit just one adenylyl cyclase isoform (5) and it remains unclear if adenylyl cyclase is a physiologically relevant direct effector for G⍺_o_ (6, 7). One hypothesis is that G⍺_o_, unlike all other animal G⍺ proteins, may not directly bind to and regulate any effector. Instead, activated G⍺_o_-GTP could signal solely by releasing Gβ*γ*, allowing the free Gβ*γ* complex to signal through its effectors, including specific Ca^2+^ and K^+^ channels (8, 9) and the synaptic vesicle fusion machinery (10). However, genetic studies in *Caenorhabditis elegans* suggest that G⍺_o_ signaling inhibits neural function by regulating its own effector(s) rather than solely *via* release of Gβ*γ* (2) and that G⍺_o_ signaling ultimately has the effect of opposing G⍺_q_ signaling (11).

Previous attempts to identify direct G⍺_o_ effectors have used yeast two-hybrid and other screening methods to identify proteins that bind G⍺_o_-GTP (12, 13, 14, 15, 16, 17, 18). Perhaps the best-characterized of these G⍺_o_ interactors are the closely related G protein–regulated inducer of neurite outgrowth (GRIN) proteins, GRIN1 and GRIN2 (14). GRIN1 binds directly to G⍺_o_ with a preference for G⍺_o_-GTP over G⍺_o_-GDP, as would be expected for an effector. In cultured neurons, GRIN1/2 act with the small G protein Cdc42 to mediate the ability of G⍺_o_ signaling to stimulate neurite outgrowth (14, 19). However, while G⍺_o_ is highly conserved in both sequence and function across animal species (20, 21), no GRIN1/2 homologs have been identified in invertebrates. In addition, there is no evidence that any candidate G⍺_o_ effector identified in these screens mediates the principal output of G⍺_o_ signaling: its ability to inhibit neurotransmitter release.

Some prior searches for G⍺_o_ effectors (14, 15, 16, 17, 18) may have been biased against finding multidomain, multisubunit, or membrane proteins, the types of molecules that serve as effectors for other G⍺ proteins. This is because the methods used relied on transforming libraries of mammalian complementary deoxyribonucleic acid (cDNA) clones into yeast or bacteria and screening for successful expression of a functional, folded protein that could interact with G⍺_o_. One very different screen for G⍺_o_ effectors used mass spectrometry (MS) to identify proteins from a *Drosophila* head extract that bound to recombinant G⍺_o_ (18). While this approach should not suffer from the same biases, it was implemented in a manner that did not measure the statistical significance of potential G⍺_o_ binding by the many proteins identified.

Here, we used immunopurification of G⍺_o_ protein complexes from mouse brain lysates followed by MS to identify RASA3 and RASA2, two GTPase-activating proteins (GAPs) for small G proteins, as G⍺_o_-interacting proteins. We show that G⍺_o_ binds directly to full-length RASA3 and that this interaction is promoted by GTP and Ca^2+^. Finally, we show that full-length RASA3 and a C-terminal fragment of RASA3 bind to G⍺_o_ in distinct fashions. Together, these experiments suggest that G⍺_o_ may bind RASA3 at two different sites and provide a possible link between G⍺_o_ signaling and small G protein signaling.

## Results

### GTP*γ*S activates G⍺_o_ in mouse brain lysates

**Figure 1:**
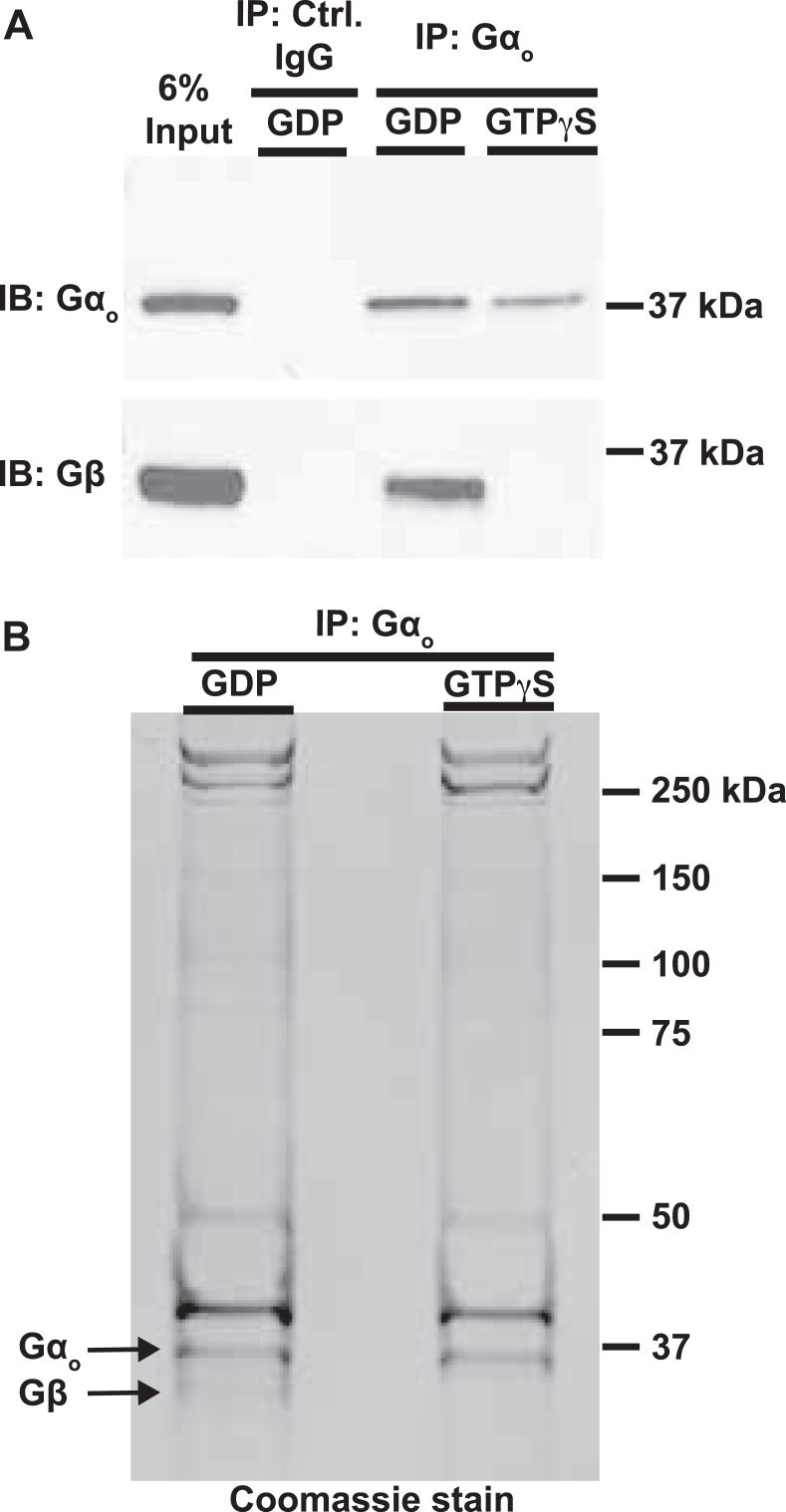
**GTPγS treatment activates G⍺_o_ in mouse brain lysates.** Lysates were pretreated with GDP or GTPγS, and G⍺_o_ protein complexes were isolated by immunoprecipitation (IP). An IgG antibody that does not recognize G⍺_o_ served as a negative control. *A*, protein complexes analyzed by immunoblot (IB). *B*, the same samples analyzed by SDS-PAGE and stained for total protein. The comparable levels of G⍺_o_ and Gβ in the immunopurified material from the GDP-treated lysate indicate that G⍺_o_ was almost entirely in inactive G⍺_o_–GDP/Gβ*γ* complexes, while absence of detectable Gβ after GTPγS treatment indicates efficient activation of G⍺_o_. GTPγS, guanosine 5′-O-[gamma-thio]triphosphate.

To identify potential effectors of G⍺_o,_ we prepared mouse whole brain lysates in buffer containing nonionic detergent to solubilize G⍺_o_ and interacting proteins. A commercially available mAb (sc-13532) efficiently immunoprecipitated G⍺_o_ from these lysates (Fig. 1*A*). When GDP was added to the lysate, G⍺_o_ appeared to be almost entirely in its inactive G⍺β*γ* heterotrimeric state, as Gβ co-immunoprecipitated with G⍺_o_ (Fig. 1*A*) in a near-stoichiometric complex (Fig. 1*B*). Direct effectors are expected to form a stable complex only with activated G⍺_o_-GTP which has dissociated from Gβ*γ*. To stably convert G⍺_o_ to this active state, we took advantage of the relatively high rate of spontaneous nucleotide exchange by G⍺_o_ (22). Pretreating the brain lysate with the very slowly hydrolyzable GTP analog, guanosine 5′-O-[gamma-thio]triphosphate (GTPγS), efficiently converted G⍺_o_ in the lysate to the active state, since Gβ was no longer detected in G⍺_o_ immunoprecipitates (Fig. 1, *A* and *B*). We performed anti-G⍺_o_ immunoprecipitations (IPs) from brain lysates at a scale that yielded several micrograms of G⍺_o_, allowing the immunopurified G⍺_o_ to be seen on gels stained for total protein (Fig. 1*B*). G⍺_o_ was one of the most abundant proteins in the immunoprecipitate, although several other proteins were also visible.

A direct effector for G⍺_o_ might be expected to co-immunoprecipitate with G⍺_o_-GTPγS but not with G⍺_o_-GDP. However, the only difference detected between the two immunoprecipitates at the level of sensitivity allowed by the total protein stain was the absence of Gβ from the G⍺_o_-GTPγS immunoprecipitate (Fig. 1*B*). Thus, if G⍺_o_ effector(s) were present specifically in the G⍺_o_-GTPγS immunoprecipitate, they were at levels sub-stoichiometric to G⍺_o_, which would not be surprising given that G⍺_o_ is one of the most abundant proteins in the brain. To identify such lower abundance proteins in the immunoprecipitates, we used MS.

### Identification of G⍺o-GTP–associated proteins by MS

To allow statistical analysis of the MS results, we performed immunopurification of G⍺_o_ protein complexes from three independently prepared mouse brain lysates. For each of these three independent biological samples, we performed three different immunopurifications: 1) purification of activated G⍺_o_ complexes from GTPγS-pretreated lysate; 2) purification of inactive G⍺_o_ from GDP-pretreated lysate; and 3) a mock immunopurification from GDP-pretreated lysates using an isotype-matched mAb that does not recognize G⍺_o_ (Fig. 1*A*). Direct G⍺_o_ effectors are expected to bind to activated G⍺_o_-GTP in preference to G⍺_o_-GDP, and the mock-purified samples served as negative controls to subtract the substantial background typical of MS results.

**Figure 2:**
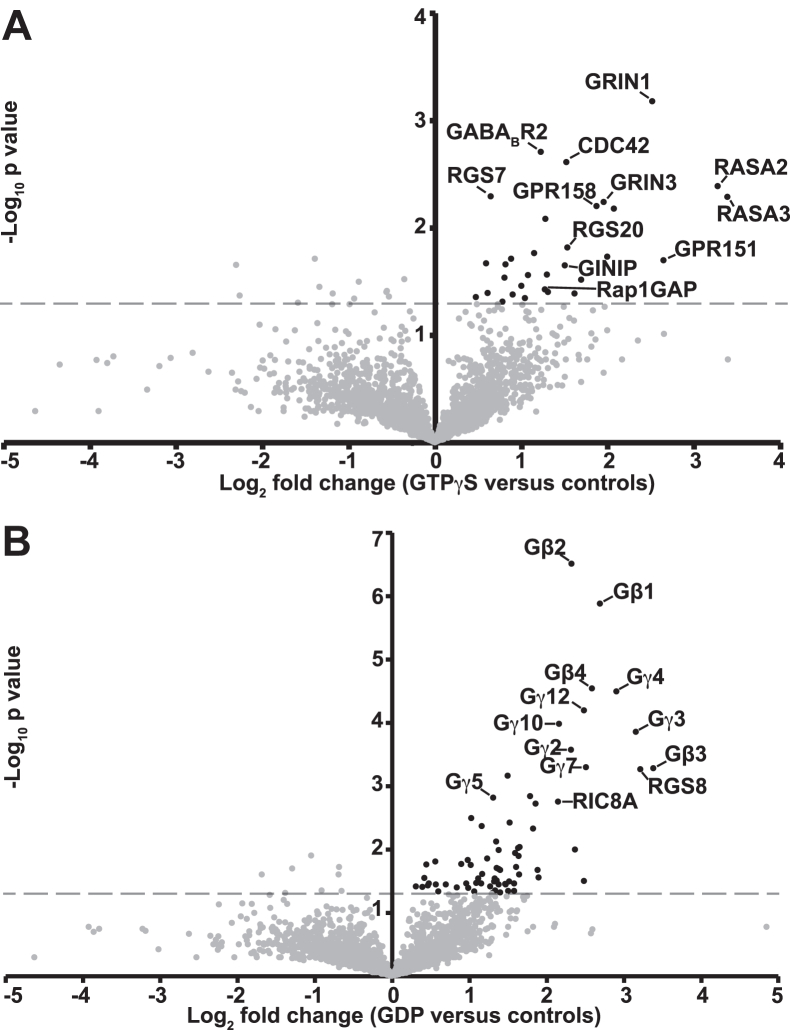
**Mass spectrometry identifies RASA2 and RASA3 as candidate G⍺_o_ effectors.***A*, volcano plot highlighting proteins enriched in immunoprecipitates of G⍺_o_-GTPγS from mouse brain lysates relative to negative controls (immunoprecipitates of G⍺_o_-GDP and immunoprecipitates generated using a control antibody that does not recognize G⍺_o_). *Dots* above the *dashed gray line* and to the *right* of the *vertical axis* represent proteins enriched in the GTPγS samples relative to these negative controls with *p* < 0.05. *B*, volcano plot in which the same data were reanalyzed to highlight proteins enriched in G⍺_o_-GDP immunoprecipitates relative to G⍺_o_-GTPγS immunoprecipitates and negative control antibody immunoprecipitates. *Dots* above the *dashed gray line* and to the *right* of the *vertical axis* represent proteins enriched in the GDP samples relative to these controls with *p* < 0.05. In (*A*) and (*B*), some of the most enriched proteins are labeled and all the enriched proteins with *p* < 0.05 are detailed in Tables S1 and S2. GTPγS, guanosine 5′-O-[gamma-thio]triphosphate; RASA, Ras GTPase-activating protein.

After MS analysis of the nine samples, we generated a volcano plot (Fig. 2*A*) to identify proteins that were enriched in the three GTPγS-treated samples relative to the six remaining samples (the three GDP-treated samples and the three mock-purified samples). Table 1 provides additional details of the MS data obtained for select protein hits. We generated a second volcano plot (Fig. 2*B*) that conversely identified proteins that were preferentially enriched in the GDP-treated samples relative to the six remaining samples. For all proteins identified as hits in these two volcano plots with *p* < 0.05, Tables S1 and S2 detail their fold enrichment just comparing the G⍺_o_-GTPγS and G⍺_o_-GDP samples (*i.e.,* leaving the mock-purified samples out of the calculation). Table S3 provides a more extensive summary of the MS results. The raw MS data are available for further analysis in the PRIDE database. *p* values shown in Figure 2 and Tables S1 and S2 were calculated using *t*-tests without correction for multiple comparisons. When correction for multiple comparisons was used, given the large number of proteins detected, none of the proteins were enriched in either the GTP*γ*S or GDP conditions at levels that were statistically significant. Nevertheless, as described in the next paragraph, many of the most enriched proteins in both the GTP*γ*S and GDP conditions were biologically significant. The biological significance of proteins in Tables S1 and S2 with higher *p* values has not been investigated.

**Table 1:** Select mass spectrometry data for GTP*γ*S-enriched proteins

Protein	RASA2	RASA3	GRIN1	CDC42	RGS20	GINIP	Rap1GAP
Fold enrichment GTP*γ*S versus GDP^a^	7.4	5.9	3.3	2.9	1.8	2.5	2.2
Fold enrichment GTP*γ*S versus GDP and antibody controls^b^	15	12	5.7	2.9	3.6	2.8	2.4
*p* value GTP*γ*S versus GDP control^a^	0.057	0.069	0.016	0.028	0.13	0.14	0.16
*p* value GTP*γ*S versus both GDP and antibody controls^b^	0.0051	0.0037	0.00067	0.0025	0.019	0.023	0.038
% sequence coverage, GTP*γ*S samples							
A	7.2	19.3	55.4	62.8	18.8	26.0	21.9
B	9.9	20.3	46.0	29.8	8.8	30.3	30.6
C	15.1	44.8	60.5	35.6	23.8	17.5	34.1
Exclusive unique peptide count, GTP*γ*S samples							
A	3	11	38	8	3	8	9
B	6	13	38	5	2	8	17
C	9	29	52	6	5	5	17
Exclusive unique spectrum count, GTP*γ*S samples							
A	3	12	46	8	4	9	9
B	6	15	48	5	2	8	18
C	9	34	66	8	5	5	20
Normalized total spectra, GTP*γ*S samples^c^							
A	2.5	9.9	41.9	8.2	3.3	7.5	7.4
B	9.3	21.3	73.2	6.7	2.7	10.7	25.3
C	9.5	35.0	76.7	8.5	4.7	4.7	18.9

a Comparing normalized total spectra for three G⍺_o_-GTP*γ*S samples *versus* three G⍺_o_-GDP samples.

b Comparing normalized total spectra for three G⍺_o_-GTP*γ*S samples *versus* six negative control samples: three G⍺_o_-GDP samples and three isotype-matched control antibody samples.

c Calculated using Scaffold software.

Our experimental methods are validated by the presence of proteins previously shown to associate with G⍺_o_-GTP or G⍺_o_-GDP among the most prominent hits in our MS results. Proteins in the upper right of Figure 2*A* preferentially copurified with activated G⍺_o_-GTPγS. GRIN1 and GRIN3 are close homologs and as purified proteins have previously been shown to bind preferentially to purified G⍺_o_-GTP *versus* G⍺_o_-GDP (14, 23). RGS20, a regulator of G protein signaling, was previously shown to co-immunoprecipitate with activated G⍺_o_ in the lysates of cultured cells transfected to express both proteins (24). Rap1GAP was found to directly bind G⍺_o_-GDP and G⍺_o_-GTP and is a potential G⍺_o_ effector (12, 13). Additional proteins highlighted in Figure 2*A* were not previously known to form stable complexes with activated G⍺_o_ but were known to be involved with heterotrimeric G protein signaling in various ways. These include the regulator of G protein signaling RGS7 and the associated G protein–coupled receptor GPR158 (25, 26, 27), and the G⍺_o_-coupled GABA_B_ receptor subunit GABA_B_R2 (28). CDC42 acts with GRIN1 to mediate G⍺_o_-induced neurite outgrowth (19). Purified G⍺ inhibitory interacting protein (GINIP) was previously shown to bind preferentially to purified G⍺_i_-GTP *versus* G⍺_i_-GDP, but did not appear to bind G⍺_o_ (29, 30). Our results extend these findings by indicating association of these other proteins with activated G⍺_o_-GTP in native brain protein complexes.

The proteins most preferentially enriched in our G⍺_o_-GDP immunopurifications were isoforms of Gβ and G*γ* subunits, further validating our MS screen (Fig. 2*B* and Table S2). The Gβ*γ* complex binds to G⍺-GDP to form the inactive G⍺β*γ* heterotrimer but dissociates from G⍺ upon activation of the G⍺ protein by exchange of GDP for GTP (3). Additional highly enriched proteins in our G⍺_o_-GDP immunopurifications included resistance to inhibitors of cholinesterase 8A and RGS8 (Fig. 2*B*), both of which function with heterotrimeric G proteins. Resistance to inhibitors of cholinesterase 8A serves as a chaperone that directly binds nascent G⍺ proteins and is released when G⍺ binds GTP (31), explaining its preferential enrichment in our G⍺_o_-GDP purifications. RGS8 has been shown to form a complex with G⍺_o_ and receptors that accelerates the rate of G protein activation (32). The list of proteins preferentially enriched in the G⍺_o_-GDP immunopurifications (Table S2) contains additional proteins not known to be associated with G⍺_o_ signaling. However, none of these additional proteins show statistical significance as strong as that of the top hits described above, and as noted in Table S2, many of these weaker hits are small guanine nucleotide–binding proteins and other proteins known to interact with them.

### Purified RASA3 binds directly to purified G⍺o-GTP in preference to G⍺o-GDP

RASA2 and RASA3 (Ras p21 protein activator 2 and 3) showed the largest enrichments of any proteins in G⍺_o_-GTPγS immunoprecipitates compared to G⍺_o_-GDP immunoprecipitates (Fig. 2*A*, Table S1), and the statistical significance of this preference was on par with that for GRIN1/3, two proteins for which direct preferential binding to G⍺_o_-GTP *versus* G⍺_o_-GDP has been well characterized (14, 23). In contrast, the interactions of RASA2/3 with G⍺_o_ have been little studied. RASA2 and RASA3 are highly similar proteins that serve as GTPase activators for small G proteins including Ras and Rap1b, thus terminating signaling by these G proteins (33, 34, 35, 36, 37). RASA3 was previously identified as a G⍺_i_-binding protein in a yeast two-hybrid screen (16). A much weaker interaction with G⍺_o_ in the same two-hybrid system was noted, and G⍺_i_ proteins were also shown to directly bind RASA3 i*n vitro* (16). Subsequently, RASA2 and RASA3 were identified as interacting partners for activated G⍺_i1_ in a proximity labeling screen (38) and as *in vitro* binding partners for recombinant G⍺_i2_ in a proteomics screen (39). For the rest of this work, we present an analysis of the interaction of purified RASA3 with purified G⍺_o_.

**Figure 3:**
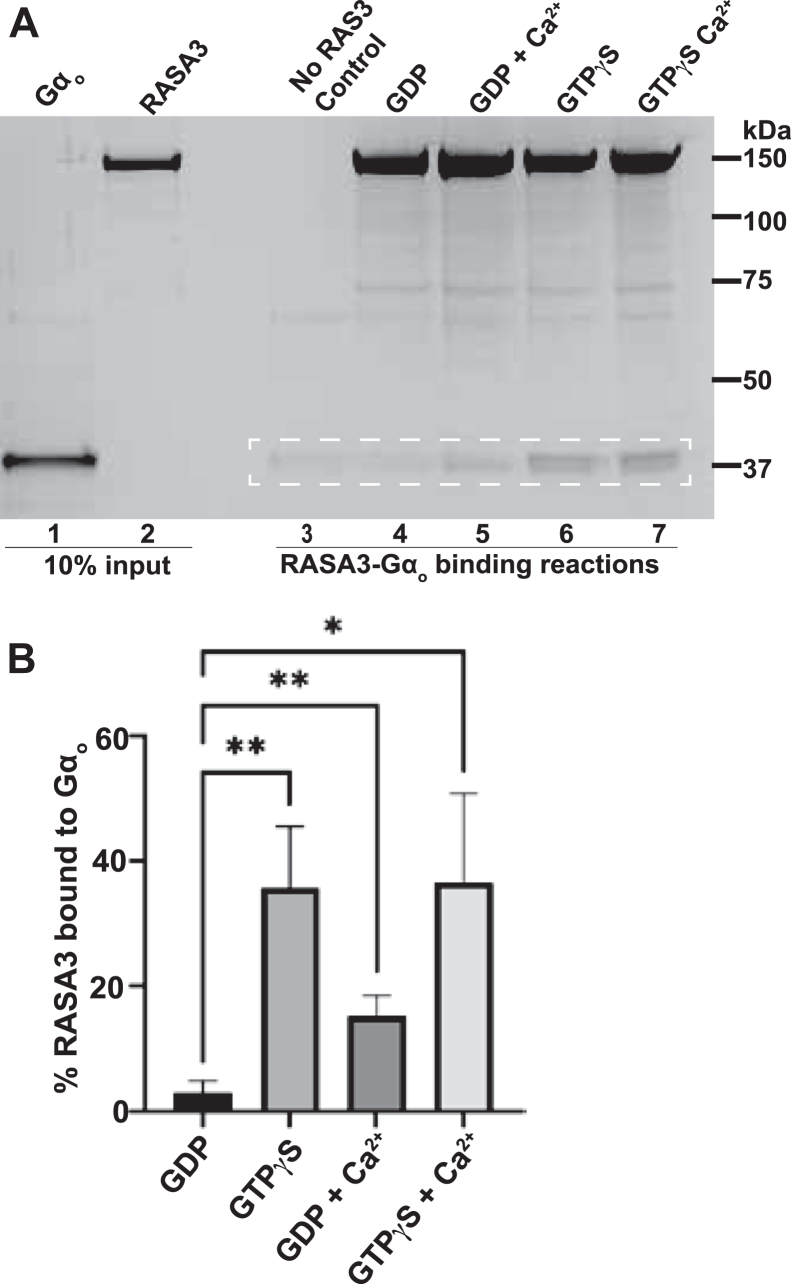
**Direct binding of purified RASA3 to G⍺_o_ is promoted by GTP*γ*S and Ca^2+^.***A*, *in vitro* binding reactions using purified full-length RASA3 and G⍺_o_. Proteins were visualized in this and other gels in this work using Coomassie R-250 dye. G⍺_o_ was prebound to GDP or GTP*γ*S and then incubated with RASA3 that had been prebound to Ni-NTA beads *via* a C-terminal His_6_ tag. Protein complexes bound to the beads after washing were analyzed by SDS-PAGE and stained with Coomassie. Ca^2+^ was added to a subset of binding reactions, as indicated. RASA3 was left out of the control reaction shown. Bands representing G⍺_o_ protein are highlighted within the *dashed white rectangle*. Ten percentage of the amounts of protein inputs into the binding reactions were loaded in lanes 1 and 2. Densitometry measurements of the known amounts of protein that formed bands in these input lanes along with densitometry measurements of the bands in the binding reaction lanes were used to determine the molar amounts of RASA and G⍺_o_ pulled down in each binding reaction. *B*, the mean percent of RASA3 bound to G⍺_o_ in each *in vitro* binding reaction condition. Error bars, standard error. ∗, *p* < 0.05, ∗∗, *p* < 0.01, unpaired two-tailed *t* test, n = 6 for GDP, n = 6 for GTP*γ*S, n = 3 for GDP Ca^2+^, n = 3 for GTP*γ*S Ca^2+^. GTPγS, guanosine 5′-O-[gamma-thio]triphosphate; RASA, Ras GTPase-activating protein.

We purified recombinant full-length human RASA3 and G⍺_o_ (Fig. 3*A*, lanes 1 and 2). RASA3 was expressed in *Escherichia coli* as an MBP-RASA3-His_6_ fusion protein with the maltose-binding protein (MBP) at its N terminus and a His_6_ tag at its C terminus, which served as affinity tags for purification and for pull-down binding assays, and to enhance solubility of RASA3. The studies presented below of “RASA3” are of this fusion protein. We also purified an MBP-His_6_ fusion protein lacking the RASA3 insertion to use as a negative control in certain experiments (Fig. S1). G⍺_o_ was expressed with affinity tags that were proteolytically cleaved off and removed during purification. Size-exclusion chromatography verified that these purified proteins were monodispersed and of the predicted molecular size.

To measure binding between these purified proteins, G⍺_o_-GDP or G⍺_o_-GTP*γ*S (1 μM) was incubated with RASA3 (0.4 μM) that had been prebound to Ni^+^-NTA resin *via* its C-terminal His_6_ tag. The resin was washed and protein complexes remaining on the resin were run on SDS-PAGE and detected with a protein stain (Fig. 3*A*). We found that RASA3 bound directly to both G⍺_o_-GDP and G⍺_o_-GTP*γ*S, with a preference for G⍺_o_-GTP*γ*S (Fig. 3*A*, compare lanes 4 and 6). We repeated this experiment six times, using independent preps of RASA3 protein for each repeat, and quantitated the results by measuring the intensity of RASA3 and G⍺_o_ bands on the gels with densitometry. We controlled for nonspecific binding of G⍺_o_ to the resin by leaving RASA3 out of the binding reactions (as shown in Fig. 3*A*, lane 3). We also controlled for possible binding of G⍺_o_ to the affinity tags on the RASA3 fusion protein by substituting the MBP-His_6_ control protein for the MBP-RASA3-His_6_ fusion protein and saw no significant binding of G⍺_o_ to this control protein (Fig. S1). Assuming one RASA3 protein binds only one G⍺_o_ protein, under our assay conditions, G⍺_o_-GDP bound to 3.0 ± 1.9% of RASA3 molecules, while G⍺_o_-GTP*γ*S bound to 35.8 ± 9.8% of RASA molecules (Fig. 3*B*). Thus, the GTP-activated conformation of G⍺_o_ had a higher binding affinity for RASA3 than did the inactive GDP-bound conformation of G⍺_o_, as would be expected if G⍺_o_ uses RASA3 as an effector. These results demonstrate that the interaction between G⍺_o_ and RASA3 that we initially detected in crude brain lysates was the result of direct binding between these two proteins.

### Calcium enhances the interaction between RASA3 and G⍺o

**Figure 4:**
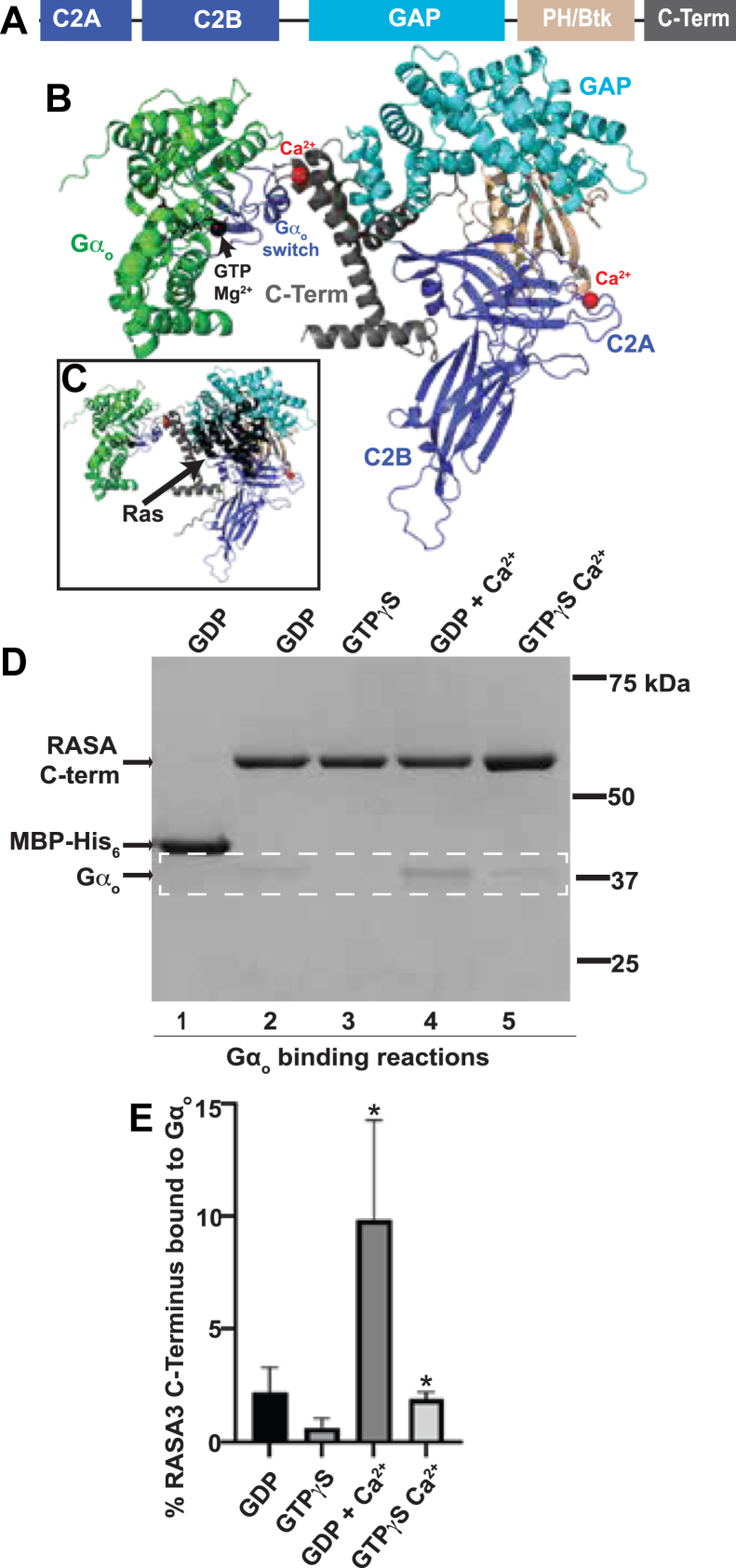
**G⍺_o_ binds directly to the C terminus of RASA3 in calcium- and nucleotide-dependent manner.***A*, predicted domains of the 834 amino acid RASA3 protein. *B*, AlphaFold 3 predicted structure of a complex between G⍺_o_ and RASA3, with RASA3 domains colored as in (*A*). The two proteins contact *via* the switch regions of G⍺_o_ (*blue*) and the predicted Ca^2+^-binding site within the C-terminal region (*dark gray*) of RASA3. A second Ca^2+^ ion bound to the C2A domain and Mg^2+^/GTP bound to G⍺_o_ are shown. *C*, the same predicted structure as in (*B*) but with Ras (*black*) shown bound to the RASA3 GAP domain as predicted by AlphaFold 3. *D*, *in vitro* binding between the C-terminal region of RASA3 and G⍺_o_. The G⍺_o_-binding assay was analogous to the one shown in Figure 3 for full-length RASA3. G⍺_o_ was prebound with GDP or GTP*γ*S and then incubated with either the control MBP-His6 protein or an MBP-(RASA3 C-terminal region)-His_6_ fusion protein (“RASA C-term”) that had been prebound to Ni-NTA beads *via* the His_6_ tag. Protein complexes bound to the beads after washing were analyzed by SDS-PAGE and stained with Coomassie. Ca^2+^ was added to a subset of binding reactions, as indicated. Bands representing G⍺_o_ protein are highlighted within the *dashed white rectangle*. The input lanes on the original gel used to analyze densitometry measurements are not shown in the image. *E*, bar graph depicting the mean percent of RASA3 C-terminal region bound to G⍺_o_ in each *in vitro* binding reaction condition. Error bars, standard error. *Asterisk*, significantly above zero (*p* < 0.05, unpaired one-tailed *t* test, n = 3). GAP, GTPase-activating protein; GTPγS, guanosine 5′-O-[gamma-thio]triphosphate; MBP, maltose-binding protein; RASA, Ras GTPase-activating protein.

RASA3 is an 834 amino acid protein with the predicted domain structure (37) illustrated in Figure 4*A*. Starting at its N terminus, it contains two C2 domains (C2A and C2B), a GAP domain, a pleckstrin homology/Bruton’s tyrosine kinase (PH/Btk) domain, and finally a 118 amino acid C-terminal region with no similarity to previously studied protein domains. C2 domains often bind Ca^2+^, and AlphaFold 3 (40) predicts the more N-terminal C2A domain binds Ca^2+^ and that there is an additional Ca^2+^-binding site in the RASA3 C-terminal region (Fig. 4*B*). Ca^2+^ binding could alter RASA3’s conformation and thus its interaction with G⍺_o_. In addition, Nafisi *et al.* (16) showed by co-IP from lysates of cultured cells that the interaction between RASA3 and the G⍺_o_ homolog G⍺_i3_ was dramatically higher in cells pretreated with a drug that activates the G⍺_q_ signaling pathway, which produces Ca^2+^ as a second messenger. The hypothesis that Ca^2+^ may change the interaction between RASA3 and G⍺_o_ is particularly intriguing because *C. elegans* genetic analysis shows that G⍺_o_ and G⍺_q_ signaling antagonize each other (2, 41), although the biochemical mechanism of this antagonism remains unclear.

To test the hypothesis that Ca^2+^ affects the interaction between G⍺_o_ and RASA3, we used the *in vitro* binding assay described above to compare binding reactions carried out in parallel with or without 2 mM Ca^2+^ added to the buffer (Fig. 3*A*). G⍺_o_-GTP*γ*S bound similar amounts of RASA3 regardless of the presence of Ca^2+^ in our binding reactions (Fig. 3*B*). However, Ca^2+^ significantly enhanced the binding between G⍺_o_-GDP and RASA3. Assuming again that one RASA3 can bind only one G⍺_o_, under our assay conditions, G⍺_o-_GDP bound 15.3 ± 3.2% of RASA3 molecules in the presence of Ca^2+^, compared to only 3.0 ± 1.9% in the absence of Ca^2+^ (Fig. 3*B*). This result provides a potential direct link between G⍺_o_ and G⍺_q_ signaling.

### The C-terminal region of RASA3 binds G⍺_o_ in a Ca^2+^-regulated manner

We used AlphaFold 3 to predict the structure of potential complexes between G⍺_o_ and RASA3 (Fig. 4*B*). The predicted structure shown has an interface predicted template modeling score of 0.86, which is considered a confident, high-quality prediction of a protein–protein interface (Abramson *et al.*,. 2024). No alternative high-quality predicted structures with different interfaces between G⍺_o_ and RASA3 were identified by AlphaFold 3. In the predicted structure, RASA3 binds to the switch regions of G⍺_o_ (Fig. 4*B*), the only regions of G⍺ proteins that change conformation depending on whether GDP or GTP is bound (4). G⍺_o_ is predicted to bind RASA3 *via* a turn between two predicted helices in the RASA3 C-terminal region. This turn is also a predicted Ca^2+^ ion–binding site. The predicted structure of the G⍺_o_/RASA3 complex shown in Figure 4*B* positions G⍺_o_ such that it does not prohibit Ras from binding to the GAP domain of RASA3 (Fig. 4*C*) in the same manner as Ras has been shown to bind the homologous domain of RasGAP (42).

To experimentally test whether the C-terminal region of RASA3 binds to G⍺_o_, we purified this region (the last 119 amino acids of RASA3) fused to an N-terminal MBP tag and C-terminal His_6_ tag, analogous to the manner in which we had expressed and purified full-length RASA3. Using the same binding assay that we used to test G⍺_o_ binding to full-length RASA3 (Fig. 3), we tested whether the RASA3 C-terminal region is sufficient to bind G⍺_o_. Figure 4*D* shows one of three replicates of this binding experiment, in which the MBP-His_6_ protein lacking the RASA3 C-terminal region served as a negative control. G⍺_o_ bound to the RASA3 C-terminal region at a level significantly above background only when Ca^2+^ was included in the binding buffer (Fig. 4, *D* and *E*), and this was true whether GDP or GTP*γ*S had been prebound to G⍺_o_. Thus, Ca^2+^ promoted binding of G⍺_o_ to the RASA3 C terminus, just as it had to the full-length RASA protein. However, the results for the RASA C terminus trended toward G⍺_o_-GDP binding more strongly than G⍺_o_-GTP*γ*S to the RASA C-terminal region, the opposite of the preference shown by the full-length RASA3, although this preference for the RASA3 C terminus was not statistically significant. A major difference between the full-length of C-terminal regions of RASA3 was that G⍺_o_ appeared to bind more strongly to full-length RASA3 than it did to the RASA3 C-terminal region: under the conditions of our binding assays, G⍺_o_ bound up to ∼35% of full-length RASA3, while G⍺_o_ bound only ∼10% of the RASA3 C-terminal reaction, despite the fact that we increased the concentrations of the proteins ≥2-fold for the C-terminal binding reactions.

We conclude that G⍺_o_ binds to the RASA3 C-terminal region, but that this binding is distinct from the binding of G⍺_o_ to full-length RASA3. Both binding interactions are promoted by Ca^2+^, but only the full-length RASA3 appears to bind preferentially and relatively strongly to activated G⍺_o_ in the absence of Ca^2+^, while the RASA3 C-terminal region can bind to G⍺_o_ in the presence of Ca^2+^, albeit relatively weakly.

## Discussion

In this study, we identified potential direct effectors for the major brain G protein, G⍺_o,_ by using immunopurification of native G⍺_o_ protein complexes from mouse brain followed by MS. We thus identified proteins that preferentially associate with active *versus* inactive G⍺_o_. The biological relevance of these findings was validated by the fact that many of the proteins identified as top hits in our MS results were previously known to directly bind G⍺_o_ and act in G⍺_o_ signaling. However, the two proteins with the strongest preference for activated G⍺_o_ in our MS results, the closely related proteins RASA2 and RASA3, had not been previously studied as potential effectors of G⍺_o_. Using purified recombinant human proteins, we showed that G⍺_o_ binds directly to RASA3, that activating G⍺_o_ with a GTP analog enhances this binding, that Ca^2+^ also enhances this binding for inactive G⍺_o_-GDP, and that the C-terminal region of RASA3 can bind G⍺_o_ with properties distinct from the G⍺_o_ binding observed for the full-length RASA3 protein. Below we discuss the implications of our results for the hypothesis that RASA2/RASA3 may act as effectors for G⍺_o_ signaling.

RASA2 and RASA3 are inhibitors of small G proteins, as they contain GAP domains similar to that of the canonical Ras inhibitor p120RasGAP (33, 34, 35, 36, 43). *In vitro*, RASA2 has GAP activity specific for Ras, while RASA3 can act on both Ras and Rap (35). Both proteins also have two C2 domains that can potentially bind Ca^2+^ and/or phospholipids, and a Ph/Btk domain. While PH domains typically bind phosphatidyl inositol membrane lipids, the PH/Btk domain of RASA3 can bind the soluble inositol phosphate Ins(1,3,4,5)P_4_, and this binding promotes RASA3 GAP activity for Ras only (34). Both RASA2 and RASA3 are expressed most strongly in the brain (33, 43), consistent with the idea that the RASA proteins might function with the neural-specific G protein G⍺_o_. The two RASA proteins may have different subcellular localizations, with RASA2 being predominantly cytoplasmic and RASA3 being constitutively associated with the plasma membrane (44). The physiological functions of RASA2 and RASA3 in the brain remain to be studied.

Our work demonstrates that full-length RASA3 binds activated G⍺_o_ in preference to inactive G⍺_o_-GDP, analogous to how all established G⍺ effectors bind their cognate G⍺ proteins (4). Structural studies show that this preference arises because the established effectors directly contact the switch regions of the G⍺ proteins, the only regions that change conformation when the G⍺ protein binds GTP. The facts that RASA2/3 G⍺_o_ are coexpressed in the brain, and that we were able to isolate native RASA/G⍺_o_ complexes from brain lysates with a binding preference for active G⍺_o_, all establish RASA2/3 as credible contenders to be effectors for G⍺_o_.

We note, however, that there is more work to do before it will be clear if and how RASA2/3 function in G⍺_o_ signaling. First, it must be established if binding to G⍺_o_ activates RASA2/3, as would be expected for a G⍺/effector interaction. G⍺_o_ might activate the GAP domain of the RASA proteins or simply recruit the RASA protein to the sites of their Ras or Rap targets on the membrane so that the GAP domain can act on these targets. Methods for preparing more stable and higher-yield preparations of purified RASA will need to be developed to carry out the biochemical studies required as part of this work. Second, cell culture and genetic studies are needed to determine if, in living neurons or intact organisms, the RASA proteins are actually required for specific aspects of G⍺_o_ signaling. Ham *et al.* (39) recently provided support for the hypothesis that activated G⍺_i2_, a G⍺ protein closely related to G⍺_o_, regulates T cell receptor signaling by sequestering RASA2 at the plasma membrane, thereby reducing the ability of RASA2 to regulate Ras in the cell interior. Because the G⍺ protein would need to be present at relatively high abundance to be effective in this model, it is intriguing to speculate that activated G⍺_o_ might similarly function by sequestering RASA2/3, since in brain, G⍺_o_ is present at a remarkably high abundance (1).

Our discovery that Ca^2+^ promotes binding of RASA3 to G⍺_o_ is interesting in light of past genetic work in *C. elegans* that demonstrates that G⍺_o_ signaling precisely opposes signaling by another G protein, G⍺_q_, which causes Ca^2+^ release (41, 45, 46, 47, 48, 49). G⍺_q_ signals by directly binding and activating two effectors: phospholipase C beta and TRIO RhoGEF (49, 50, 51). Phospholipase C beta activity hydrolyzes the phosphoinositide membrane lipid PIP_2_ to generate the soluble inositol phosphate second messenger IP_3_, which can be converted to Ins(1,3,4,5)P_4_ and also causes release of cytosolic Ca^2+^. The RASA2/3 proteins contain PH/Btk domains that bind phosphoinositides and inositol phosphates (34), C2 domains (52) that may bind Ca^2+^, and the C-terminal domain of RASA3, as demonstrated in our work, appears to have an additional Ca^2+^-binding site. If RASA2 and RASA3 function in G⍺_o_ signaling, these observations provide a number of possible biochemical mechanisms by which G⍺_q_ and G⍺_o_ signaling might interact to antagonize each other. Because we showed that Ca^2+^ alters the interaction of RASA3 with G⍺_o_, the hypothesis that Ca^2+^ release induced by G⍺_q_ signaling might alter G⍺_o_ signaling through RASA3 is particularly worth considering. The second G⍺_q_ effector, TRIO RhoGEF, activates the small G protein Rho. RASA2/3 have been shown to inactivate the small G proteins Ras and/or Rap. While the RASA and Trio RhoGEF proteins have opposite effects on small G protein signaling, they apparently have specificity for different small G proteins, so it is unclear if or how a possible G⍺_o_–RASA2/3 signaling pathway could antagonize G⍺_q_–TRIO RhoGEF signaling.

Our work suggests RASA3 may have two different binding sites for G⍺_o_: one on the full-length RASA protein that preferentially binds G⍺_o_-GTP over G⍺_o_-GDP and a second on the RASA3 C-terminal region that is weaker and may show a preference for G⍺_o_-GDP. Binding of G⍺_o_ to both the full-length RASA3 protein and to the C-terminal region of RASA3 is enhanced by Ca^2+^. There are three possible Ca^2+^-binding sites on RASA3 that might influence binding to G⍺_o_: one on each of the two C2 domains and a third on the C-terminal domain site predicted by AlphaFold 3. A speculative model is that G⍺_o_ might bind to the full-length RASA3 protein in a manner that activates the RASA3 GAP domain to carry out downstream signaling. In this model, G⍺_q_ signaling could antagonize this G⍺_o_ signaling by causing Ca^2+^ release, which would alter the RASA3/G⍺_o_-binding interaction in a manner that changes G⍺_o_ signaling. When G⍺_o_ hydrolyzes GTP to GDP, thus moving to the inactive conformation, G⍺_o_ might physically move to bind its second site on the RASA3 C terminus. This configuration might continue to hold G⍺_o_, perhaps allowing for extended signaling by free Gβ*γ*, preventing reassociation of G⍺_o_-GDP with Gβ*γ* to terminate all signaling as might otherwise occur. Such an effect would be analogous to the proposed effect of G⍺ inhibitory interacting protein on signaling initiated by the G protein G⍺_i_ (29). Future work to test the above model would need to determine if Ca^2+^ binding to the various domains of RASA3 is required for the observed effects of Ca^2+^ on the *in vitro* interactions of RASA3 with G⍺_o_, if these effects occur at physiological concentrations of Ca^2+^, and if they are functionally significant for G⍺_o_ signaling *in vivo*.

## Experimental procedures

### Immunopurification of G⍺_o_ protein complexes from mouse brain lysates

Mouse brain lysates were prepared as previously described (53). Briefly, whole adult mouse brain lysates were homogenized in buffer [20 mM Hepes pH 7.4, 25 mM potassium acetate, 320 mM sucrose, 1% Triton X-100, and protease inhibitors (aprotinin, benzamidine, chymostatin, leupeptin, PMSF, and pepstatin A)], incubated on ice for 30 min, centrifuged at 100,000*g* for 1 h, and the supernatants were collected. Protein concentration was quantified by Bradford assay (Bio-Rad), and aliquoted lysates were snap frozen for storage at −80 °C until use. For the immunopurification reactions, 30 μg of mouse anti-G⍺_o_ (Santa Cruz Biotechnology, sc-13532) or a negative control mouse IgG (Santa Cruz Biotechnology, sc-69786) was crosslinked to protein A/G agarose beads using the Pierce crosslink IP kit (Thermo Fisher Scientific, 26147). For each antibody, 10 μl of packed beads were washed three times with 1× coupling buffer (diluted with water from 20× stock in the Pierce crosslink IP Kit) and then incubated for 2 h with 30 μg antibody at room temperature. The bead–antibody complex was then washed three times with 1× coupling buffer. Fifty microliters of Pierce Crosslink IP kit DSS crosslinker working solution was added, and the complex was incubated for 1 h at room temperature. The bead–antibody complex was washed three times with Pierce crosslink IP kit elution buffer, which contains a primary amine, followed by two washes with IP buffer [25 mM Tris, 150 mM NaCl, 1 mM EDTA, 1% NP-40, 5% glycerol, pH 7.2]. Mouse brain lysates were diluted to 5 mg/ml, and MgCl_2_ was added to 10 mM and GDP or GTP*γ*S were added to 100 μM. Four hundred microliters of each lysate sample was incubated with 10 μl of protein A/G agarose beads at 4 °C for 1 h on a rotator to preclear it. Each precleared lysate was mixed with antibody-crosslinked beads and incubated for 2 h at room temperature on a rotator. Beads were than washed three times with IP buffer followed by one time with Pierce crosslink IP kit conditioning buffer (diluted to 1× with water from 100× stock), moved to a new tube, and all buffer was removed. Fifty microliters of 2× LDS buffer (Thermo Fisher Scientific, NP0007; diluted from 4× with water) was added and the tubes were incubated at 55 °C for 30 min. The supernatant was then transferred to a new tube, 5% β-mercaptoethanol (BME) was added, and samples were incubated for another 15 min at 55 °C before analysis. Immunopurification reactions were performed in 500 μl Pierce spin columns (Thermo Fisher Scientific, 69705).

### SDS-PAGE and immunoblotting

For SDS-PAGE, 40 μl of the IP samples or proteins from *in vitro* binding reactions were run on NuPage 4-12% Bis-Tris gels (Thermo Fisher Scientific) in Mops buffer (Thermo Fisher Scientific, NP0001). Gels to be Coomassie stained were washed in water for 15 min, incubated overnight in Imperial protein stain (Thermo Fisher Scientific, 24615), and destained in water for at least 1 h. Gels were imaged on a BioRad ChemiDoc imaging system. Images were processed in ImageJ (https://imagej.net/ij/). For immunoblots, proteins from SDS-PAGE gels were transferred to nitrocellulose membranes and membranes were probed with mouse anti-Gβ (Santa Cruz Biotechnology, sc-166123) followed by horseradish peroxidase anti-mouse (abcam, ab131363 or rabbit anti-G⍺_o_) (54) followed by horseradish peroxidase–linked anti-rabbit (Bio-Rad, 5196-2504). Membranes were treated with Super Signal West Pico/Femto Maximum Sensitivity substrate to visualize the protein (Thermo Fisher Scientific, 34580). The Western blot analysis shown in Figure 1*A* was repeated for each mouse brain lysate sample subjected to analysis by MS with similar results: strong Gβ signal was detected in every G⍺_o_ immunoprecipitate after pretreatment of the lysate with GDP, but the Gβ signal was always reduced to near-background levels. Quantitation of the Western blots indicates that GTP*γ*S pretreatment of mouse brain lysates activated 98 ± 1% of G⍺_o_ in these lysates.

### Mass spectrometry

IP samples were run ∼1 cm into 4 to 12% Bis-Tris gels. The ∼1 cm of each gel lane containing an entire IP sample was cut from the gel and analyzed by MS. Gel pieces were fixed with a 45% methanol, 45% water, 10% acetic acid mixture and washed three times with water followed by a 50% acetonitrile (ACN)/50%100 mM ammonium bicarbonate solution. Gel pieces were than reduced with 4.5 mM DTT at 37 °C for 20 min followed by a 20 min acylation step at room temperature in the dark with 10 mM iodoacetamide. Samples were then washed for 20 min with 50% ACN/50% 100 mM ammonium bicarbonate followed by another 20 min in 50% ACN/50% 25 mM ammonium bicarbonate. Gel pieces were dried in a speed vac for 10 min and treated with trypsin (Promega Trypsin Gold MS grade, diluted 200-fold). Trypsin digestion took place overnight at 37 °C. Digested peptides were extracted by soaking for 15 min in 80% ACN/20% water with 0.1% TFA. Extracted peptides were dried in a speed vac and stored in the −20 °C until LC-MS/MS analysis. LC-MS/MS was run on a Q-Exactive Plus Mass Spectrometer (Thermo Fisher Scientific) equipped with a Waters nanoACQUITY UPLC system. Data-dependent acquisition was carried out. Peptides were trapped in a Waters Symmetry UPLC column and were separated in a 1.7 μM, 75 mm × 250 mM nanoACQUITY UPLC column. To ensure a high level of identification and quantitation integrity, a resolution of 70,000 was utilized for MS and 20 tandem mass spectrometry (MS/MS) spectra (17,500 resolution) were acquired per MS scan using higher-energy collision dissociation (HCD) with normalized collision energy of 28. All MS (profile) and MS/MS (centroid) peaks were detected in the Orbitrap. Trapping was carried out for 3 min at 5 μl/min in 99% buffer A (0.1% formic acid in water) and 1% buffer B [(0.075% formic acid in ACN] prior to eluting with linear gradients set to reach 5% B at 2 min, 25% B at 140 min, 40% B at 165 min, and 90% B at 170 min and then remain at 90% B for 10 min prior to dropping back to 3% B at 182 min and continuing to run for 18 min. Dynamic exclusion was set for a 30 s window with charge state exclusion of unassigned, 1+, 7+, 8+, >8+ and peptide match preferred. Minimum automatic gain control for MS/MS was set at 1000. Four blank injections (two 100% ACN, one at 50% ACN, and one just buffer A) followed each injection to ensure against sample carry over. MS quality control included Hela and transferrin digest injections. Raw data files were processed, and peaks were picked utilizing Proteome Discoverer 2.4 (ThermoFisher Scientific, https://thermo.flexnetoperations.com). A SwissProt *Mus musculus* protein database was used to identify proteins. Search conditions were set as follows: two missed cleavages were allowed; variable modifications included oxidation (M), acetyl (K), phospho (SY), and diglycine (K); fragment tolerance 0.02 Da and parent tolerance 10 PPM.

Scaffold software (Proteome Software, version 4.11.1, https://www.proteomesoftware.com) was used to analyze MS data and generate volcano plots. Across the nine samples analyzed, a total of 151,555 spectra were identified at 95% minimum confidence with 0.01% decoy false discovery rate. A total of 2540 proteins were detected at 99.9% minimum confidence by at least two peptides with a 0% decoy false discovery rate. Total normalized spectra count was used to generate fold change values. Tables S1 and S2 show fold change values between the GTP*γ*S and the GDP conditions; however, to generate the Figure 2 volcano plots, the isotype-matched antibody control was included as an additional control when generating fold change values. The minimum signal value was set to 0.5.

### DNA constructs

Recombinant human G⍺_o_ was expressed from an *Escherichia coli* plasmid with an N-terminal glutathione S-transferase (GST) tag, followed by a His_6_ tag and a tobacco etch virus (TEV) protease cleavage site, generating plasmid pHSB6. A human RASA3 cDNA was cloned into a derivative of plasmid pMAL-c2 (New England Biolabs, NEB) to generate a plasmid (pHSB3) that expresses a fusion protein consisting of N-terminal MBP followed by a TEV cleavage site, full-length RASA3, and a C-terminal His_6_ tag (pHSB4). To express the RASA3 C-terminal regions, the last 354 nucleotides of the RASA3 cDNA was cloned into pET-29b construct to which an N-terminal MBP tag followed by a TEV cleavage site had been added (pHSB5). All constructs were sequenced to confirm the correct plasmid structure and sequence.

### Purification of G⍺_o_

GST-His6-TEV-G⍺_o_ was expressed in *E. coli* BL21 cells. Cells were grown in 1 L broth, shaking at 37 °C to an absorbance at 600 nm (*A*_*600*_) of 0.6 and induced with 50 μM (IPTG to promote expression of the protein. Cells were grown for a further 16 to 18 h at 16 °C and collected. Bacterial cell pellets were resuspended in 50 ml lysis buffer (50 mM Hepes pH 7.2, 150 mM NaCl, 1 mM EDTA, 1 mM DTT, 1 μM GDP, and protease inhibitors (Thermo Fisher Scientific, A32963)) and incubated with lysozyme (10 mg/ml) on ice for 20 min. Cells were lysed by sonication on ice (30 s sonication followed by 1 min rest, repeated 5 times). Ten percentage of glycerol was added to the lysate and insoluble material was pelleted. Soluble material was passed through a 0.45 μM filter and mixed with 2 ml of packed glutathione sepharose resin (Thermo Fisher Scientific, 16100) prewashed with lysis buffer, for 1 h at 4 °C, rotating end-over-end. The resin was washed 2× with lysis buffer, 2× with lysis buffer containing 11 mM MgCl_2_ and 2 mM ATP, and 2× with HED buffer (50 mM Hepes pH 8, 1 mM EDTA, 1 mM DTT) containing 1 μM GDP. All washes used buffer at 5× the bead volume. G⍺_o_ protein was cleaved off the resin with His_6_-tagged TEV protease (New England Biolabs). For TEV cleavage, 250 units of His-TEV was added to 2 ml of HED buffer and incubated with the beads for 3 h at room temperature rotating end-over-end. After centrifugation, the supernatant, containing cleaved G⍺_o_ protein, was mixed with 100 μl of packed Ni-NTA resin (Qiagen, 30210) to remove the His-TEV and any GST-His6 or full-length GST-6His-TEV-G⍺_o_ or present. For most experiments, the G⍺_o_ protein in solution was concentrated to 0.5 ml using a Pierce protein concentrator (PES, 10K MWCO) and run over a Superdex 200 Increase 10/300 GL size-exclusion column (Cytiva) using HED buffer not containing any nucleotide to isolate monodispersed G⍺_o_ and remove free GDP. Protein concentration was determined by Bradford assay (Bio-Rad). The protein was aliquoted, snap-frozen in liquid nitrogen, and was stored at −80 °C. The yield of purified G⍺_o_ was typically 0.5 mg per liter of culture.

### Expression and purification of RASA3 proteins

RASA3 constructs were expressed in *E. coli* BL21 cells using the same protocol described above for the expression of G⍺_o_. Because purified RASA3 was sensitive to freeze/thaw, the protein was purified repeatedly and used the day of purification without freezing. Typical culture volumes were 500 ml and volumes provided below are for this volume of culture. Bacterial cell pellets were resuspended in 25 ml lysis buffer PBS (American Bio 10× concentrate, AB11072, diluted to 5×), adding 10 mM BME and protease inhibitors (Thermo Fisher Scientific, A32963), and incubated with lysozyme (10 mg/ml) for 20 min at 4 °C. Cells were lysed by sonication as described above. Ten percentage of glycerol and 0.25% Triton X-100 were added to the lysate and incubated for 20 min at 4 °C. Insoluble material was pelleted by centrifugation and the supernatant was passed through a 0.45 μM filter. The lysate was incubated with 0.5 ml of packed Ni-NTA resin (Qiagen, 30210) for 30 min at 4 °C, rotating end-over-end. The lysate–resin mixture was transferred to a column and washed with 10 ml lysis buffer containing 10% glycerol, 0.25% Triton X-100, and 25 mM imidazole followed by another wash with the 10 ml of same buffer lacking Triton X-100 and glycerol. Protein was eluted in 1 ml lysis buffer with 250 mM imidazole. Eluted protein was diluted 10-fold in amylose resin buffer (50 mM sodium phosphate pH 7.4, 150 mM NaCl, 10 mM BME, protease inhibitors) and bound to a column containing 0.75 ml amylose resin. Bound protein was washed with 15 ml amylose resin buffer followed by 10 ml amylose resin buffer containing 10% glycerol. Protein was eluted (4 × 0.5 ml fractions) in amylose resin buffer containing 10% glycerol, 0.25% Triton X-100, and 10 mM maltose. Protein was concentrated from 2 ml to 0.5 ml using a Pierce protein concentrator PES, (30K MWCO for full-length RASA3 or 10K MWCO for the C-terminal region of RASA3). In most experiments, the entire protein pool was run over a Superdex 200 increase 10/300 GL size-exclusion column (Cytiva) to verify that monodispersed protein was isolated. The final protein storage buffer was 50 mM sodium phosphate pH 7.4, 150 mM NaCl, 10 mM BME, protease inhibitors, 10% glycerol, and 0.25% Triton X-100. Concentration was determined by Bradford assay (Bio-Rad).

### Expression and purification of MBP-His control

An MBP-His_6_ construct (Addgene plasmid 38066) was expressed in BL21 cells and purified following the same methods described above for RASA3 constructs.

### AlphaFold structural predictions

Protein complex structures were predicted using the AlphaFold server (alphafoldserver.com) with the following inputs: human G⍺_o_ sequence, human RASA3 sequence, two GTP, two Mg^2+^, and four Ca^2+^.

### *In vitro* binding assays

G⍺_o_ (0.5 μM for experiments done with full-length RASA3 or 1 μM for experiments done with the C-terminal region of RASA3) was incubated with 10 μM of GDP or GTP*γ*S in binding buffer (50 mM Hepes pH 8, 150 mM NaCl, 10 mM BME, 10 mM MgCl_2_) for 1 h at 30 °C to promote nucleotide exchange. Five hundred microliters of 0.25 μM of purified MPB-RASA3-His_6_ protein or 250 μl of 1 μM of purified MBP-RASA3 C-tail-His_6_ protein in binding buffer was incubated with 15 μl of packed Ni-NTA resin prewashed with binding buffer in separate 0.5 ml low-protein binding microcentrifuge tubes (Thomas Scientific, 1147A17) for 1 h at room temperature, rotating end-over-end. CaCl_2_ was added to 2 mM for binding assays that included calcium. Beads were washed three times with 12× the bead volume of binding buffer (plus 2 mM CaCl_2_ where appropriate). The 500 μl of 0.5 μM G⍺_o_ or 250 μl of 1 μM G⍺_o_ solution that had been preincubated with GDP or GTP*γ*S was added to each reaction, adding 25 mM imidazole from a 1.5 M stock and 2 mM CaCl_2_ from a 1 M stock where appropriate and allowed to incubate for 1 h at room temperature. Beads were then washed three times with 12× bead volume of binding buffer plus 25 mM imidazole. On the last wash, beads were moved to a new tube with a cut pipette tip to avoid elution of any proteins that nonspecifically bound to the tube. Fifty microliters of 2× LDS buffer (Thermo Fisher Scientific, NP0007) with 5% BME was added and reactions were incubated at 70 °C for 20 min. Forty microliters of each sample were analyzed by SDS-PAGE. Purified MBP-His_6_ protein or beads with no MBP fusion protein added served as the negative controls.

For each binding experiment, all samples to be compared, along 10% of the RASA3 protein and 10% of the G⍺_o_ input into each binding reaction, were run in separate lanes of the same gel. After Coomassie staining, the intensity of each RASA3 and G⍺_o_ protein band was determined by densitometry, and the molar amounts of these proteins in each pull-down lane were determined by comparison to the known amounts of these proteins in the 10% input lanes. This allowed a calculation of the percent of the pulled-down RASA3 that was bound to G⍺_o_ in each binding reaction.

## Data availability

The mass spectrometry proteomics data have been deposited to the ProteomeXchange Consortium *via* the PRIDE (55) partner repository with the dataset identifier PXD069453.

## Supporting information

This article contains supporting information (Fig. S1 and Tables S1, S2, and S3).

## Conflicts of interest

The authors declare that they have no conflicts of interest with the contents of this article.
