# Supplementary material for: Binding of the brain G protein G⍺o to its potential effector RASA3 is promoted by Ca2+
Source: J Biol Chem. 2025 Dec 3;302(1):110999. doi: 10.1016/j.jbc.2025.110999 (PMC12796736; doi:10.1016/j.jbc.2025.110999)
Supplement: Figure S1 [file mmc1.pdf]

Figure S1

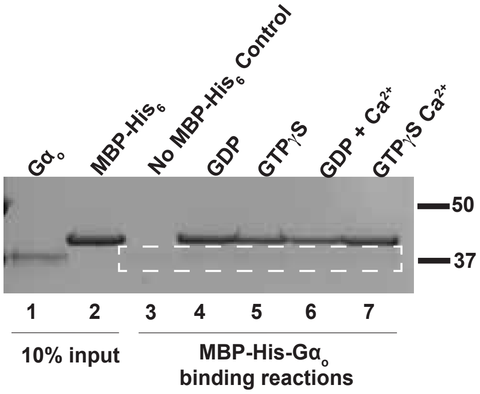

Figure S1. The MBP-His6 affinity tags do not detectably bind to Gα_o. *In vitro* binding reactions using purified Gα_o protein (lane 1) and a purified control protein consisting of just the MBP-His$_6$ affinity tags (lane 2). This binding assay is analogous to the one shown in Figure 3. Gα_o was pre-bound to GDP or GTPγS and then incubated with MBP-His$_6$ that had been prebound to Ni-NTA beads via its C-terminal His$_6$ tag. Protein complexes bound to the beads after washing were analyzed by SDS-PAGE and stained with Coomassie. Ca^{2+} was added to a subset of binding reactions, as indicated. MBP-His$_6$ was left out of the control reaction shown in lane 3. The area where bands representing Gα_o protein might be present are highlighted within the dashed white rectangle. In the experiment shown here and in other replicates, the G$\alpha_o$signals in lanes 4-7 were similar to the background in lane 3, indicating no significant binding of G$\alpha_o$to MBP-His$_6$ under the conditions of these assays.
