# Supplementary material for: Binding of the brain G protein G⍺o to its potential effector RASA3 is promoted by Ca2+
Source: J Biol Chem. 2025 Dec 3;302(1):110999. doi: 10.1016/j.jbc.2025.110999 (PMC12796736; doi:10.1016/j.jbc.2025.110999)
Supplement: Table S1 [file mmc2.docx]

**Table S1 Proteins enriched in G⍺_o_-GTP pulldowns**

| Protein name^a^ | Protein name, long version | Fold enrichment in GTP𝛾S versus GDP samples^b^ | p value GTP𝛾S versus controls^c^ |
| --- | --- | --- | --- |
| GRIN1 | G protein regulated inducer of neurite outgrowth 1 | 3.3 | 6.7E-05 |
| GABR2 (GABA_B_R2) | Gamma-aminobutyric acid type B receptor subunit 2 | 2.1 | 0.002 |
| CDC42 | Cell division control protein 42 homolog | 2.9 | 0.0025 |
| RASA2 | Ras GTPase-activating protein 2 | 7.4 | 0.0041 |
| RGS7 | regulator of G protein signaling 7 | 1.9 | 0.0051 |
| RASA3 | Ras GTPase-activating protein 3 | 5.9 | 0.0052 |
| GRIN3 | G protein regulated inducer of neurite outgrowth 3 | 2.0 | 0.0058 |
| MGLYR (GPR158) | Metabotropic glycine receptor (G protein coupled receptor 158) | 2.4 | 0.0063 |
| NECP1 (NECAP1) | Adaptin ear-binding coat-associated protein 1 | 4.1 | 0.0066 |
| AFG32 (AFG3L2) | Mitochondrial inner membrane m-AAA protease component AFG3L2 | 2.7 | 0.0083 |
| RGS20 | Regulator of G-protein signaling 20 | 1.8 | 0.015 |
| SYK (LysRS) | Lysyl-tRNA synthetase | 2.0 | 0.017 |
| GPR151 | G-protein coupled receptor 151 protein | 4.0 | 0.019 |
| ARK72 | Aflatoxin B1 aldehyde reductase member 2 | 1.7 | 0.019 |
| RB11A (RAB11A) | Ras-related protein Rab-11A | 6.3 | 0.02 |
| EPHA6 | Ephrin type-A receptor 6 | 1.5 | 0.022 |
| GRIP1 | Glutamate receptor-interacting protein 1 | 1.9 | 0.022 |
| PHF24 (GINIP) | **PHD finger protein 24 (**Gα inhibitory interacting protein) | 2.5 | 0.023 |
| KIT | Mast/stem cell growth factor receptor Kit | 2.3 | 0.028 |
| SC6A1 (GAT-1) | Sodium- and chloride-dependent GABA transporter 1 (GABA transporter 1) | 1.9 | 0.028 |
| IF4A1 (eIF4A1) | Eukaryotic initiation factor 4A-I | 1.7 | 0.029 |
| KCNA3 | Potassium Voltage-gated channel subfamily A member 3 | 3.9 | 0.031 |
| OST48 (DDOST) | Dolichyl-diphosphooligosaccharide- protein glycosyltransferase non-catalytic subunit | 1.6 | 0.034 |
| RPGP1 (Rap1GAP) | Rap1 GTPase-activating protein 1 | 2.2 | 0.038 |
| GRM2 (mGluR2) | Metabotropic glutamate receptor 2 | 1.6 | 0.04 |
| GNAZ (G⍺_z_) | Guanine nucleotide-binding protein G⍺_z_ subunit | 1.4 | 0.041 |
| TEN3 | Teneurin-3 | 4 | 0.041 |
| TM9S2 | Transmembrane 9 superfamily member 2 | 1.3 | 0.042 |
| Syt2 | Synaptotagmin 2 | 1.3 | 0.045 |
| VPS41 | Vacuolar protein sorting-associated protein homolog 41 | 2.1 | 0.045 |

**Table S1 Continued**

| Protein name^a^ | Protein name, long version | Fold enrichment in GTP𝛾S versus GDP samples^b^ | p value GTP𝛾S versus controls^c^ |
| --- | --- | --- | --- |
| COL12 (CL-12) | Collectin-12 | 2.1 | 0.045 |
| NDUAD (NDUFA13) | NADH dehydrogenase [ubiquinone] 1 alpha subcomplex subunit 13 | 2.1 | 0.045 |
| PSA5 | Proteosome subunit alpha type 5 | 1.6 | 0.049 |

a Protein names used in UniProtKB (The UniProt Consortium, 2025) followed in some cases in parentheses by an alternative commonly used name.

b Calculated by Scaffold software comparing the average number of peptide spectra in the three G⍺_o_-GTP𝛾S samples versus three G⍺_o_-GDP samples.

c Calculated by Scaffold for comparing the average number of peptide spectra in the three G⍺_o_-GTP𝛾S samples versus the six negative control samples (three G⍺_o_-GDP samples and three isotype matched control antibody samples).
