# Supplementary material for: Binding of the brain G protein G⍺o to its potential effector RASA3 is promoted by Ca2+
Source: J Biol Chem. 2025 Dec 3;302(1):110999. doi: 10.1016/j.jbc.2025.110999 (PMC12796736; doi:10.1016/j.jbc.2025.110999)
Supplement: Table S2 [file mmc3.docx]

**Table S2 Proteins enriched in G⍺_o_-GDP pulldowns**

| Protein name^a^ | Protein name, long version | Fold enrichment in GDP versus GTP𝛾S samples^b^ | p value GDP versus controls^c^ |
| --- | --- | --- | --- |
| Gβ2 | G protein beta 2 subunit | 3.9 | 3.2E-07 |
| Gβ1 | G protein beta 1 subunit | 5.2 | 1.4E-06 |
| Gβ4 | G protein beta 4 subunit | 4.7 | 2.9E-05 |
| G𝛾4 | G protein gamma 4 subunit | 4.9 | 3.3E-05 |
| G𝛾12 | G protein gamma 12 subunit | 3.5 | 6.5E-05 |
| G𝛾10 | G protein gamma 10 subunit | 4.5 | 0.00011 |
| G𝛾3 | G protein gamma 3 subunit | 5.9 | 0.00014 |
| G𝛾2 | G protein gamma 2 subunit | 3.3 | 0.00027 |
| G𝛾7 | G protein gamma 7 subunit | 3.9 | 0.00052 |
| Gβ3 | G protein beta 3 subunit | 6.8 | 0.00054 |
| RGS8 | Regulator of G protein signaling 8 | 7.7 | 0.00056 |
| LPGAT1 | Lysophosphatidylglycerol acyltransferase 1 | 2.9 | 0.0007 |
| UBR3 | N-recognin E3 ubiquitin ligase 3 | 2.9 | 0.0015 |
| G𝛾5 | G protein gamma 5 subunit | 2.5 | 0.0016 |
| RIC-8A | Resistance to inhibitors of cholinesterase 8A | 2.3 | 0.0018 |
| FHIP1B^e^ | FHF complex subunit HOOK interacting protein 1B | 3 | 0.0019 |
| ARL1^d^ | ADP-ribosylation factor-like protein 1 | 2.1 | 0.0033 |
| DRG2^d^ | Developmentally-regulated GTP-binding protein 2 | 3.2 | 0.0039 |
| DCAKD | Dephospho-CoA kinase domain containing | 2.9 | 0.0043 |
| ADGRL2^e^ | Adhesion G protein-coupled receptor L2 | 2.7 | 0.0048 |
| SYRM (RARS2) | Probable arginine--tRNA ligase, mitochondrial | 2.6 | 0.0077 |
| Transportin-2^e^ | Transportin-2 | 1.8 | 0.0093 |
| Cyclin-Y | Cyclin-Y | 2.1 | 0.0097 |
| 5MP1^e^ | Eukaryotic initiation factor 5 mimic protein 1 | 3.3 | 0.01 |
| Galectin-related protein | Galectin Related Protein | 2.2 | 0.01 |
| Importinβ1^e^ | Importin beta 1 | 1.8 | 0.012 |
| eIF4𝛾3 | Eukaryotic translation initiation factor 4 gamma 3 | 2.4 | 0.013 |
| LONP1 | Lon protease homolog (mitochondrial) | 1.8 | 0.014 |
| GBRG2 | GABA_A_ receptor subunit gamma-2 | 1.6 | 0.015 |
| CAMKV | **CaM kinase-like vesicle-associated protein V** | 1.4 | 0.016 |
| IMP4 | **Importin 4** | 1.9 | 0.017 |

**Table S2 continued**

| Protein name^a^ | Protein name, long version | Fold enrichment in GDP versus GTP𝛾S samples^b^ | p value GDP versus controls^c^ |
| --- | --- | --- | --- |
| Tubulin ⍺4A^d^ | Tubulin alpha 4A subunit | 1.2 | 0.017 |
| ADA23 (ADAM23) | Disintegrin and metalloproteinase domain-containing protein 23 | 1.5 | 0.018 |
| RGS10 | Regulator of G protein signaling 10 | 3 | 0.019 |
| SV2-related^d^ | Synaptic vesicle 2 related protein | 2.1 | 0.02 |
| Imp7^e^ | Importin 7 | 1.5 | 0.021 |
| 2A5A (PP2A-B56⍺) | Serine/threonine-protein phosphatase 2A 56 kDa regulatory subunit alpha isoform | 2.6 | 0.021 |
| TBCD^e^ | Tubulin-specific chaperone D | 3.3 | 0.022 |
| Sec61B^e^ | Protein transport protein Sec61 subunit beta | 1.7 | 0.025 |
| PTPRN | Receptor-type tyrosine-protein phosphatase-like N | 2.9 | 0.025 |
| Imp11^e^ | Importin 11 | 2.4 | 0.028 |
| TPPP^d^ | Tubulin polymerization-promoting protein | 1.4 | 0.029 |
| DLGP3 (SAPAP3) | Disks large-associated protein 3 | 3 | 0.029 |
| MMS19 | MMS19 nucleotide excision repair protein homolog | 2.4 | 0.029 |
| Stathmin-2^e^ | Stathmin-2 | 2.1 | 0.032 |
| G⍺_14_ | G protein alpha subunit 14 | 5.6 | 0.032 |
| CUL2 | Cullin-2 | 1.7 | 0.033 |
| SCFD2 | Sec1 family domain-containing protein 2 | 2.6 | 0.033 |
| Metaxin-1^e^ | Metaxin-1 | 1.6 | 0.035 |
| MTCH2 | Mitochondrial carrier homolog 2 | 1.9 | 0.035 |
| TOM70 | Import receptor of the translocase complex of the outer mitochondrial membrane (TOM) | 1.8 | 0.035 |
| RNA cyclase | RNA 3'-terminal phosphate cyclase | 1.7 | 0.035 |
| Tubulin ⍺1A^e^ | Tubulin ⍺1A | 1.2 | 0.035 |
| Calpain-5 | Calpain-5 | 1.9 | 0.035 |
| srGAP1^e^ | SLIT-ROBO Rho GTPase-activating protein 1 | 1.5 | 0.036 |
| Imp-5^e^ | Importin 5 | 1.7 | 0.036 |
| - DBT | **Lipoamide acyltransferase E2 component of branched-chain alpha-keto acid dehydrogenase complex, mitochondrial** | 1.4 | 0.036 |
| UBE3c | **Ubiquitin-protein ligase E3C** | 1.8 | 0.036 |
| GAPDH | Glyceraldehyde-3-phosphate dehydrogenase | 1.3 | 0.038 |
| MAPRE2 | **Microtubule-associated protein RP/EB family member 2** | 1.2 | 0.039 |

**Table S2 continued**

| Protein name^a^ | Protein name, long version | Fold enrichment in GDP versus GTP𝛾S samples^b^ | p value GDP versus controls^c^ |
| --- | --- | --- | --- |
| RASGRP2^e^ | RAS guanyl-releasing protein 2 | 1.8 | 0.039 |
| Tubulin ⍺1C^e^ | Tubulin ⍺1C | 1.2 | 0.04 |
| FADS2 | Acyl-CoA 6-desaturase | 1.3 | 0.041 |
| ATP5PD | **ATP synthase peripheral stalk subunit d, mitochondrial** | 1.7 | 0.041 |
| - EXC6B | Exocyst complex component 6B | 1.7 | 0.045 |
| CAMLG | Guided entry of tail-anchored proteins factor CAMLG | 2.2 | 0.046 |
| G⍺_olf_^d^ | G protein G(olf) alpha subunit | 1.9 | 0.046 |
| AAA1 (ASC1) | **Asc-type amino acid transporter 1** | 2.1 | 0.047 |
| UGT8 | **2-hydroxyacylsphingosine 1-beta-galactosyltransferase** | 2.1 | 0.036 |
| SAC1 | Phosphatidylinositol-3-phosphatase (homolog of yeast Sac1) | 1.7 | 0.048 |
| Exportin-2^e^ | Exportin-2 | 1.5 | 0.049 |

a Protein names used in UniProtKB (The UniProt Consortium, 2025) or a more commonly-used alternative name.

b Calculated by Scaffold software comparing the total normalized number of peptide spectra in the three G⍺_o_-GDP samples versus the three G⍺_o_-GTP𝛾S samples.

c Calculated by Scaffold comparing the total normalized number of peptide spectra in the three G⍺_o_-GDP versus the six negative control samples (three G⍺_o_-GTP𝛾S samples and three isotype matched control antibody samples).

d Guanine nucleotide-binding protein

e Binds to a guanine nucleotide-binding protein
